# Endangered Frogs Coexist with Fungus Once Thought Fatal

**DOI:** 10.1371/journal.pbio.0020389

**Published:** 2004-10-05

Amphibian declines have reached crisis proportions in various parts of the world. In many areas, habitat loss is the likely culprit. But when mass die-offs suddenly occurred in relatively undisturbed habitats, the cause was far less obvious. Fourteen species suffered either extinctions or major declines in the pristine rainforests of Queensland, Australia, between 1979 and 1993. It was suggested in 1996 that some unknown disease had spread through the populations, but no pathogen was discovered until 1998, when the fungus Batrachochytrium dendrobatidis was identified from sick and dead frogs. Since then, several lines of evidence suggest that B. dendrobatidis may be involved in frog declines: the fungus has been found on frogs in afflicted areas; lab studies show that it's highly pathogenic to some frog species; and pathological evidence links it to host mortality. But with little information about the prevalence of this fungal infection in wild frogs, or how the disease impacts frogs in the wild, the causal role of this chytrid fungus remains unclear.

To evaluate the effects of B. dendrobatidis on frogs in their natural habitat, Richard Retallick et al. focused on six species living in the high-elevation rainforest streams of Eungella National Park in Queensland, Australia, where frog losses were “particularly catastrophic.” Two species vanished between 1985 and 1986: the Eungella Gastric-Brooding Frog (Rheobatrachus vitellinus), which is now thought extinct, and the Eungella Torrent Frog (Taudactylus eungellensis), which later reappeared in a few small populations. Other local frog species escaped this period relatively unscathed.

**Figure:**
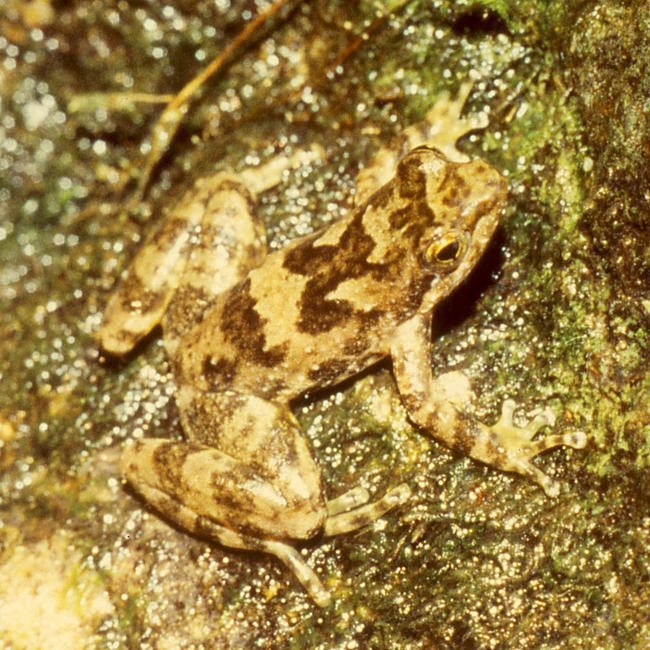
Taudactylus eungellensis (Photo: Richard Retallick)

Retallick captured frogs from six sites from 1994 to 1998, clipped one or two toe tips from each frog to age and identify them, and then released the frogs back into the wild. At the time, B. dendrobatidis had yet to be identified, but Retallick retained the toe tips, and the authors tested the toes for disease in 2002–2003. Fungal infections were found in two species—T. eungellensis and Litoria wilcoxii/jungguy (the latter consists of two species that are indistinguishable without genetic analysis); the other four species were infection-free. L. wilcoxii/jungguy did not decline to any great extent during the 1985–1986 die-off.

The proportion of infected T. eungellensis frogs was greatest at three particular sites, which showed peak infections during cooler months. Prevalence of infection was highest during winter and spring, but did not vary from year to year, suggesting that the infection is now endemic. Fungal infections were found in 27.7% of L. wilcoxii/jungguy frogs, with no evidence that prevalence differed among sites, seasons, or individuals (males, females, or subadults). The probability of recapture was significantly lower for frogs that were already infected when first captured. While this might suggest a correlation between infection and death, it's impossible to distinguish death from simple failure to recapture the animal. On further analysis, McCallum and colleagues found no evidence that survival differed between infected and uninfected frogs, suggesting that this potentially devastating amphibian disease now coexists with the frogs, with little effect on their populations.

These results, the authors conclude, “show unequivocally” that remaining populations of T. eungellensis, a rainforest frog listed as endangered, “now persist with stable infections of B. dendrobatidis.” While these findings do not exonerate the fungus as the agent of mass declines, they do rule out the possibility that the fungus caused the decline, then vanished from the area, allowing frog populations to recover. The authors allow that it's possible that B. dendrobatidis did not cause the initial T. eungellensis declines. Or alternately, the fungus could have emerged as a novel pathogen in the ecosystem, causing massive casualties before some form of evolutionary response took hold. Surviving frog populations may have evolved resistance to the pathogen, for example, or less virulent strains of the fungus may have evolved. If it turns out that frog populations can develop resistance to the chytrid fungus, the researchers point out, then a conservation program of captive breeding and selecting for resistance could potentially thwart the extinction of these, and other, critically endangered frogs. A critical next step, then, is to determine whether frogs and fungus do coevolve.

